# Intractable Vomiting as an Initial and Evasive Presentation of Neuromyelitis Optica Spectrum Disorder

**DOI:** 10.7759/cureus.87410

**Published:** 2025-07-07

**Authors:** Sally Wirrom-Jorrie, Anara Karaca, Carlos Canessa

**Affiliations:** 1 General Internal Medicine, King's College Hospital NHS Foundation Trust, London, GBR

**Keywords:** elderly medicine, neuroinflammatory disease, neurological autoimmune disorders, neurology, neuromyelitis optica spectrum disorder (nmosd)

## Abstract

Neuromyelitis optica spectrum disorder (NMOSD) is an uncommon autoimmune neurological condition that is rarely associated with vomiting as the only initial symptom. We report a 67-year-old Caribbean female who was admitted to the general medicine ward with intractable vomiting for about two weeks. Her condition two weeks later progressed to brainstem syndrome, progressive dysphagia, and slurred speech. A month from admission, she developed quadriparesis, symptoms initially suggestive of a stroke. The underlying neurological diagnosis remained unclear for some time. Her initial symptoms were attributed to viral gastroenteritis, later with gastroscopy to esophagitis and subsequently to a possible stroke, which brain CT and MRI ruled out. However, a brain MRI with contrast revealed longitudinal and extensive hyperintense T2 changes in the brainstem, including the area postrema (lower medulla) and the cervical region. Ultimately, after more than a month of her admission, she was diagnosed with NMOSD, confirmed by the presence of aquaporin-4 antibodies in the cerebrospinal fluid via lumbar puncture. She received high-dose corticosteroid therapy and plasma exchange as acute treatment, followed by maintenance with mycophenolate. This led to improvements in her speech and swallowing; however, the improvement in her muscle weakness was not that prominent. After a long hospital admission, a patient was transferred to a neurorehabilitation centre to support recovery and enhance muscle strength, along with immunosuppressive treatment. During her extended hospital stay, the patient required nasogastric feeding, and her course was complicated by recurrent chest and urinary tract infections. This case highlights the atypical presentation and diagnostic challenges of NMOSD. We hope this report will help clinicians consider NMOSD earlier in similar clinical scenarios.

## Introduction

Vomiting is a nonspecific symptom in neurological conditions; however, the emergence of subsequent neurological signs should prompt further investigation with neuroimaging, such as brain and spinal MRI [1,2]. This case highlights the importance of maintaining a broad differential diagnosis, including rare neurological disorders, notably when stroke has been excluded.

Neurological causes of dysarthria, dysphagia, and paresis commonly include stroke, demyelinating disorders, and neuromuscular myopathies; this constellation localises in the lower brainstem [3-6].

Neuromyelitis optica spectrum disorder (NMOSD) is a rare demyelinating condition. It typically involves demyelination of the optic nerves and spinal cord, leading to symptoms such as visual loss, limb weakness, and myelopathy, associated with bladder and bowel dysfunction [7]. However, the brainstem syndrome presentation of NMOSD, such as vomiting, dysarthria, dysphagia, facial palsy, and paresis, is an uncommon manifestation, leading to diagnostic challenges and delays in the treatment.

## Case presentation

A 67-year-old Caribbean female presented with a two-week history of vomiting and general malaise. During her initial self-referral to the emergency department, she was treated with antiemetics such as ondansetron and discharged with a presumed diagnosis of viral gastroenteritis. However, she re-presented two days later with intractable vomiting 20 times a day; was found to have diabetic ketosis without acidosis, glucose of 21.1 mmol/L, ketones of 2.5 mmol/L, and normal pH in gas; and was admitted to the general medicine ward.

Her medical history included type 2 diabetes mellitus on metformin treatment, a non-smoker, and no history of alcohol abuse.

On the ward, she continued to vomit and had severe electrolyte imbalances requiring potassium IV replacement. Two weeks after admission to the general medicine ward, she developed dysarthria and progressive dysphagia in both solids and liquids. CT and MRI brain scans ruled out acute stroke. With the input of the speech and language therapy and nutrition teams, she was started on a 12-hour nutrition feeding regimen through a nasogastric tube (NG).

More than a month after admission, she developed quadriparesis, with the condition more pronounced on the left side. Muscle power was 0-1/5 on the left upper and lower limbs, 3-4/5 on the right upper limb, and 3/5 on the right lower limb. Patchy sensory findings and involvement of the dorsal column were also noted. Additionally, the patient developed bladder and bowel dysfunction, needing a urine catheter and bedpans. A repeat brain CT and MRI excluded an acute stroke.

Cranial nerves, including CN VII, IX, X, and XII, were affected; the rest of the CN, including II, III, IV, and VI, involving the eyes, were intact.

**Figure 1:**
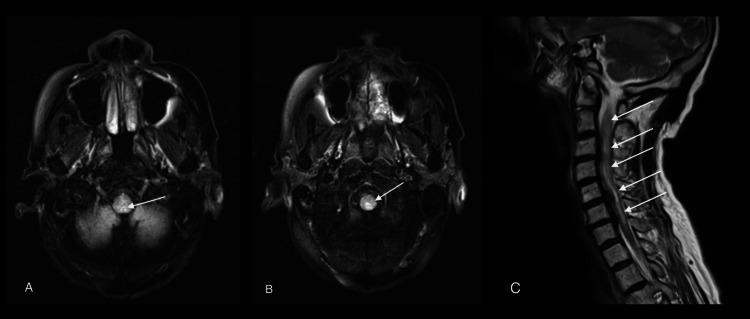
MRI T2 FLAIR brain axial and sagittal views (A) Visualisation of the lower medulla (brainstem) at the level of the cerebellum. Note a hyper-attenuated lesion situated at the dorsal medulla with increased T2 signal and mild diffuse and heterogeneous enhancement FLAIR: Fluid attenuated inversion recovery (B) Axial views of cervical cord with increased T2 signals with heterogeneous enhancement (C) Sagittal views of cervical cord with diffuse hyperintense T2 up to C7 MRI: magnetic resonance imaging; FLAIR: fluid-attenuated inversion recovery

However, the contrast brain MRI, requested due to suspicion of a demyelinating cause, revealed longitudinally extensive contrast-enhancing signal change from the lower medulla to C7, with mild cord expansion and involvement of the area postrema (Figures 1A, 1B). The contrast spinal MRI showed longitudinally extensive and contrast-enhancing signal changes from the obex to C7, with mild cord expansion and involvement of the area postrema (Figure 1C). We consulted a neuroradiologist, who suggested considering NMOSD and checking for aquaporin-4 antibody (AQP4-IgG). The consulted neurology team requested a lumbar puncture (LP) and initiated high-dose corticosteroid therapy after the LP, suspecting a demyelinating process. The patient received 1 g methyl prednisolone IV for five days. 

Meanwhile, extensive investigations done within a month of her admission, including vitamin B12, folate, thyroid function, HIV and syphilis serology, lupus anticoagulant, antinuclear antibody (ANA), anti-neutrophil cytoplasmic antibody (ANCA), anti-ds DNA, creatine kinase, and anti-neuronal antibodies, were either negative or unremarkable, as shown in Table 1.

**Table 1:** All laboratory results ALT: alanine transaminase; ALP: alkaline phosphatase; AST: aspartate aminotransferase; CRP: C-reactive protein

Variables	Values	Reference Range
White blood cells	12.7 x 10^9^/L	(2.9-9.6)
Hemoglobin	134 g/L	(115-148)
Neutrophil	9.84 x 10^9^/L	(1.5-6.1)
Urea	10 mmol/L	(2.5-7.8)
Creatinine	70 mmol/L	47-99)
Sodium	137 mmol/L	(135-145)
Potassium	3.3 mmol/L	(3.5-5.3)
Calcium	2.13 mmol/L	(2.2-2.6)
ALP	79 U/L	(30-130)
ALT	7 U/L	<55
AST	14 U/L	<34
CRP	7 mg/L	<5
Erythrocyte sedimentation rate	74 mm/hr	<20
B12	487 ng/L	(182-692)
Folate	3.8 ug/L	(3.1-20.5)
Thyroid-stimulating hormone	0.99 mIU/L	(0.35-4.94)
Free thyroxine	14.5 pmol/L	(9-19)
Creatinine kinase	63 U/L	(25-200)
Anti-nuclear antibody (ANA)	Negative	
Anti-dsDNA	<0.5 IU/mL	<10
Anti-neutrophil cytoplasmic antibodies (ANCA)	<0.2 IU/L	<0.35
Lupus anticoagulant	1%	<1.1
Anti-neuronal Hu	Negative	
Anti-neuronal Ri	Negative	
Anti-neuronal Yo	Negative	
Myelin oligodendrocyte glycoprotein antibodies	Negative	
Oligoclonal banding in cerebrospinal fluid (CSF)	Negative	
Anti-aquaporin-4 CSF	Positive	
Anti-ganglioside ab CSF	Negative	
Treponema total antibody	Negative	
Anti-HIV	Negative	

Gastroscopy revealed esophagitis, and an abdominal CT scan showed no significant pathology.

After pulse steroid treatment, no visible change was noted, and with neurology team input, a five-day course of plasma exchange (PLEX) was initiated but interrupted due to a catheter-associated urinary tract infection requiring intravenous antibiotics. She could complete four sessions of PLEX, followed by the maintenance treatment with mycophenolate. These interventions led to significant improvement in her dysphagia, allowing removal of the NG and resumption of oral intake, as well as improved speech.

A couple of weeks later, we received results from LP; multiple sclerosis, acute demyelinating neuropathy, and myelin oligodendrocyte glycoprotein (MOG) disease were ruled out, as both oligoclonal bands, ganglioside antibodies, and anti-MOG were negative in the cerebrospinal fluid (CSF). However, AQP4-IgG was found to be positive in CSF, confirming a diagnosis of NMOSD, as shown in Table 1.

Notably, her vision remained unaffected throughout her illness. Despite these gains, her muscle weakness in the upper and lower limbs showed only a mild improvement, as well as patchy sensory changes. After a prolonged hospital stay, she was transferred to a neurorehabilitation centre to aid in functional recovery and muscle strengthening in immunosuppressive maintenance treatment.

During an extended hospital stay, she developed complications such as aspiration pneumonia and urinary catheter infections, which were managed with intravenous antibiotics.

## Discussion

Our case highlights diagnostic challenges of rare neurological disorders with nonspecific presentations, such as intractable vomiting; a sudden development of dysarthria and dysphagia within two weeks; and quadriparesis mimicking stroke at first glance, two more weeks later, but with the presence of patchy sensory signs. We need to keep in mind demyelinating causes such as multiple sclerosis, Guillain-Barré syndrome, NMOSD, and myelin oligodendrocyte glycoprotein-associated disease (MOGSD), which were subsequently investigated in our case.

Note that these findings developed over time, making diagnosis difficult.

The findings of acute brainstem syndrome, such as vomiting, dysarthria, dysphagia, vertigo, unsteadiness, and dizziness, are one of the supporting criteria for the NMOSD 2015 diagnostic criteria. The other supporting requirements include area postrema syndrome (i.e., lower brainstem (intractable vomiting and hiccups)), acute optic neuritis, and acute myelitis [8].

Intractable vomiting as a presenting symptom in this case is caused by involvement of the area postrema, as this area, adjacent to the fourth ventricle, contains chemoreceptors that can trigger vomiting [9].

In addition to brainstem syndrome, our patient's brain and spinal contrast MRI demonstrated a hyperintense lesion at the lower medulla and cervical demyelination, and CSF demonstrated elevated AQP4-IgG, which met the diagnostic criteria for NMOSD [8].  Please note that AQP4-IgG can also be requested in serum without doing LP, but in our case, we did LP to rule out other differentials mentioned above.

Initially, the most common presentations of NMO in literature were longitudinally extensive transverse myelitis (59%) and optic neuritis (17%) [10]. However, recently, more cases with brainstem presentations have been reported, and the discovery of positive AQP4-IgG led to a change in the diagnostic criteria in 2015 by the NMO panel. Brainstem syndrome, though it is less common by 14%, is one of the diagnostic criteria of NMOSD. Equally, AQP4-IgG in serum or CSF is a highly specific (>98%) and sensitive (64%) biomarker, and its discovery has greatly aided the diagnosis of NMOSD as well [8].

In another series of NMOSD cases with brainstem syndrome and AQP4-IgG, there were broader brainstem presentations such as dysarthria, diplopia, vertigo, facial weakness, ataxia, and quadriparesis [11]. Our patient seems to present with area postrema syndrome, which eventually developed to broad brainstem involvement, as mentioned above, but without optic neuritis.

Some other atypical manifestations and associations with other autoimmune diseases in NMOSD have been reported as rare events. Still, they may represent a broader spectrum of symptoms, such as intractable pruritus, cutaneous manifestations, Horner syndrome, or paroxysmal sneezing [12-15].

Mainly, contrast brain MRI and AQP4-IgG in serum or CSF are diagnostic tests for NMOSD.

Treatment can be divided into acute and maintenance remissions. Acute treatment includes pulse steroid treatment and plasmapheresis; maintenance treatment involves immunosuppression with mycophenolate, rituximab, and other immunosuppressants [16,17].

Some patients may additionally need rehabilitation due to their long recovery, like our patient, probably because of her extensive demyelination from the lower medulla to the C7 cervical region.

## Conclusions

This case is essential to remind clinicians that brainstem and/or area postrema syndrome can mimic gastroenteritis and stroke, as in this case, and delay the diagnosis of this rare condition. Thus, by raising awareness about atypical presentations of NMOSD, such as vomiting and stroke-like symptoms, we would emphasise the importance of prompt brain contrast MRI imaging, along with serum and/or CSF for AQP4-IgG, for early diagnosis and treatment of similar cases in the future.
